# Syntheses, Characterization, and Photo-Hydrogen-Evolving Properties of Tris(2,2'-bipyridine)ruthenium(II) Derivatives Tethered to an H_2_-Evolving (2-phenylpyridinato)platinum(II) Unit

**DOI:** 10.3390/molecules15074908

**Published:** 2010-07-14

**Authors:** Masayuki Kobayashi, Shigeyuki Masaoka, Ken Sakai

**Affiliations:** Department of Chemistry, Faculty of Science, Kyushu University, 6-10-1 Hakozaki, Higashi-ku, Fukuoka 812-8581, Japan

**Keywords:** solar energy conversion and storage, hydrogen energy, photocatalysis, platinum(II) catalysts, ruthenium complexes, molecular devices

## Abstract

With the aim of developing new molecular devices having higher photo-hydrogen-evolving activity, Pt(ppy)ClX units (ppy = 2-phenylpyridinate, X = Cl^-^ or DMSO; DMSO = dimethylsulfoxide) have been employed as an H_2_-evolving site, as the catalytic activity of [Pt(ppy)Cl_2_]^-^ was confirmed to be higher than those of other mononuclear platinum(II) complexes. In the present study, two new heterodinuclear Ru(II)Pt(II) complexes, produced by condensation of [Ru(bpy)_2_(5-amino-phen)]^2+^ (bpy = 2,2'-bipyridine, phen = 1,10-phenanthroline) with [Pt(cppy)Cl_2_]^-^ and Pt(cppy)(DMSO)Cl (cppy = 9-carboxy-phenylpyridinate), respectively, have been prepared and their photo-hydrogen-evolving activities have been evaluated in detail. The ineffectiveness of these systems as photo-hydrogen-evolving molecular devices are interpreted in terms of their negative driving forces for the photoinduced electron transfer from the triplet MLCT excited state of the Ru chromophore to the π*(ppy) orbital of the catalyst moiety.

## 1. Introduction

**Scheme 1:**
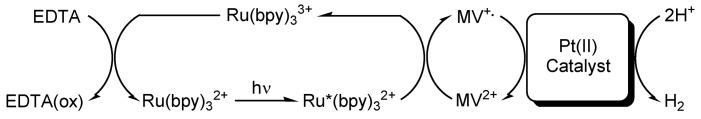
Three-component system for H_2_-evolving cycle.

Visible light-induced water splitting into dihydrogen and dioxygen (2H_2_O + 4hν → 2H_2_ + O_2_) has attracted considerable attention towards the development of artificial photosynthesis generating renewable energy from sunlight. Around the beginning of 1980s, tris(2,2'-bipyridine)ruthenium(II) ([Ru(bpy)_3_]^2+^) was extensively investigated due to its potential application as a photosensitizer driving these water splitting reactions [1,2,3,4,5,6]. In extended studies in this area, our group has made continuous efforts in the last two decades to better understand the hydrogen evolution reaction (HER) from water catalyzed by Pt(II)-based molecular catalysts by employing the so-called “three-component system”, which consists of EDTA (ethylenediaminetetraacetic acid disodium salt) as a sacrificial electron donor, [Ru(bpy)_3_]^2+^ as a photosensitizer, and methyl viologen (*N*,*N’*-dimethyl-4,4’-bipyridinium, abbreviated as MV^2+^); as an electron relay (Scheme 1) [7,8,9,10,11,12]. 

Important findings so far may be summarized as follows: (i) amidate-bridged *cis*-diammineplatinum(II) dimers [Pt(II)_2_(NH_3_)_4_(*μ*-amidato)_2_]^2+^ (amidate = α-pyridonate, α-pyrrolidinonate, acetamidate, 2-fluoroacetamidate) [7,8,9] with a shorter Pt-Pt distance display higher H_2_-evolving activity in comparison with those having a longer Pt-Pt distance; (ii) the mononuclear Pt(II) complexes having negatively charged chloride ligands, e.g., *cis*-PtCl_2_(NH_3_)_2_, PtCl_2_(ethylenediamine), and PtCl_2_(4,4’-dicarboxy-bpy), show substantially higher activity than those ligated with only neutral ligands, e.g., [Pt(NH_3_)_4_]^2+^ and [Pt(bpy)_2_]^2+^, even though their activities are still lower in comparison with those of the dinuclear Pt(II) complexes [10]; (iii) the Pt(II) compounds having electron acceptor ligands, e.g., those tethered to viologen type moieties, exhibit higher activity than those without such ligands, regardless of the Pt nuclearity [11]; (iv) the catalytic activity is lowered when the axial sites of the Pt(II) coordination plane are sterically hindered with regard to the access of a hydrogen atom [10,11]; (v) the destabilization of the HOMO which often corresponds to the filled Pt(II) d_z_2 orbital results in the catalytic enhancement [7,8,9,12].

**Scheme 2:**
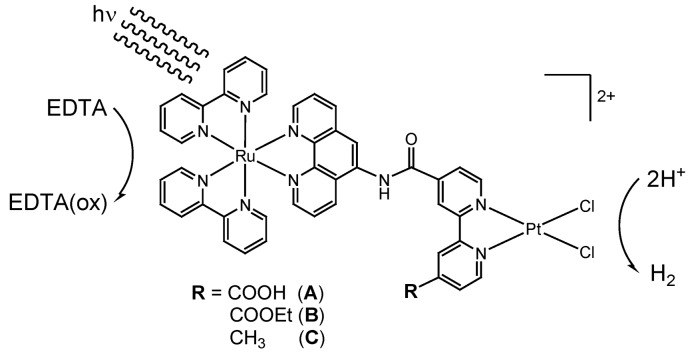
Covalently linked single-component system.

During the above studies, we were also successful in inventing the first active model of a ‘photo-hydrogen-evolving’ molecular device (**A**; R = COOH; see Scheme 2), which is capable of driving photoreduction of water by EDTA into H_2_ as a single-molecular photocatalyst [13]. Some other examples of such hybrid molecules serving both as a photosensitizer and an H_2_-evolving catalyst were also reported by several other researchers [14,15,16,17,18,19,20,21]. We further undertook extended studies on the Ru(II)Pt(II)-based photo-hydrogen-evolving molecular devices [22,23,24], in which several important concepts have been developed as follows: (vi) the photoinduced HER driven by **A** was ascertained to proceed via a bimolecular pathway, which was suggested to involve the formation of a diplatinum intermediate [13,22]; (vii) the HER driven by **A** was shown to obey saturation kinetics as a function of the EDTA concentration, revealing that an ion-pair adduct of the dicationic **A** and the dianionic form of EDTA (dideprotonated form YH_2_^2-^, where EDTA = YH_4_) is a key intermediate [22]; (viii) it was also realized that the driving force for the photoinduced electron transfer from the ^3^MLCT excited state of the [Ru(bpy)_2_(phen)]^2+^ moiety to the π*(bpy) orbital attached to the Pt(II) ion, leading to the HER, can be rationally controlled by changing the substituent group R (see Scheme 2; **A**-**C**) [23,24]. 

**Scheme 3:**
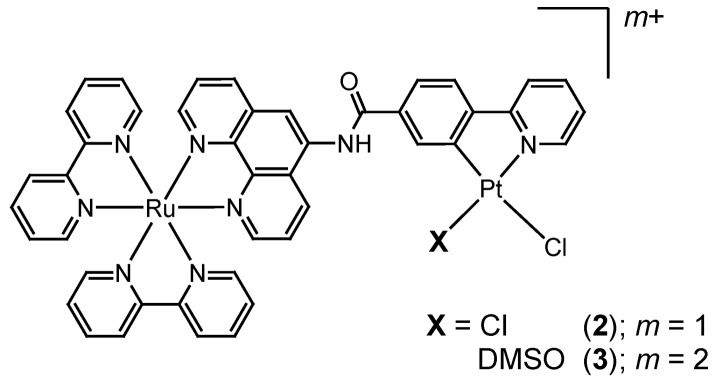
Chemical structures of the new Ru(II)Pt(II) dimers investigated.

In order to improve the photocatalytic efficiency of these photo-hydrogen-evolving molecular devices, it is important for us to develop new systems by replacing the Pt(II)-based H_2_-evolving unit of **A**-**C** with a more highly active catalyst. In this context, we previously examined the H_2_-evolving activity of (2-phenylpyridinato)platinum(II), [Pt(ppy)Cl_2_]^-^ (Hppy = 2-phenylpyridine), in the hope of destabilizing the Pt(II) d_z_2 orbital by the ligation of a strong σ-donating carbanion ligand. This has a relevance to the above-mentioned structure-activity relationship (v) [12]. In the study, we ascertained that [Pt(ppy)Cl_2_]^-^ indeed exhibits higher H_2_-evolving activity in comparison with the Pt(bpy)Cl_2_ derivatives. In this study, we have synthesized a new type of photo-hydrogen-evolving molecular device by replacing the Pt(bpy)Cl_2_ moiety in **A** with a [Pt(ppy)Cl_2_]^-^ derivative (**2**) (Scheme 3). We have also prepared an analog of **2** (*i.e.*, **3**) in which one of the two chloride ions bound to the Pt(II) ion of **2** is replaced by a DMSO molecule; Pt(ppy)(DMSO)Cl (DMSO = dimethylsulfoxide). Although these systems have been found to be ineffective towards the HER as a single-molecular device, the present study further ascertains that the driving force for the intramolecular electron transfer must be accurately controlled in the development of such photosynthetic molecular devices.

## 2. Results and Discussion

### 2.1. Syntheses

**Scheme 4:**
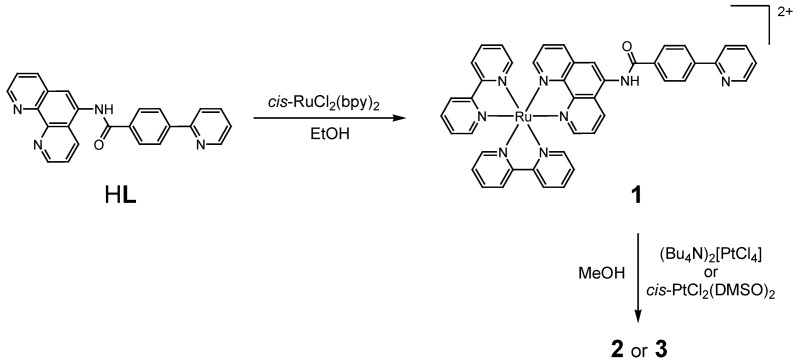
Synthetic routes to complexes **1**-**3**.

The new Ru(II)Pt(II) dimers **2** and **3** were prepared according to Scheme 4. In the first step, *cis*-RuCl_2_(bpy)_2_ was reacted with *N*-(1,10-phenanthrolin-5-yl)-4-(2-pyridyl)benzamide (H**L**) [25] in ethanol, giving complex ligand **1** in a high yield (85%). In this reaction, none of an undesirable ruthenium complex ligated with a phenylpyridinate chelate in H**L** was produced, which was confirmed by ^1^H-NMR and ESI-TOF MS. In the second step, the monoruthenium precursor **1** was platinated by either (Bu_4_N)_2_[PtCl_4_] (Bu_4_N^+^ = tetra(*n*-butyl)ammonium) or *cis*-PtCl_2_(DMSO)_2_ in methanol to give the corresponding Ru(II)Pt(II) dimer (*ca*. 40% for **2** and *ca*. 70% for **3**), where the synthetic method and the yield of **3** are similar to those previously reported for Pt(ppy)(DMSO)Cl [26]. An important feature is that the use of a pressure-resistant vial allowed us to carry out the synthesis at a relativity high temperature (140 °C), which is much higher than the boiling point of methanol under the ambient pressure (65 °C). The target Ru(II)Pt(II) dimers have been characterized by elemental analysis, ESI-TOF MS, IR, and ^1^H-NMR.

### 2.2. Spectroscopic study

**Figure 1:**
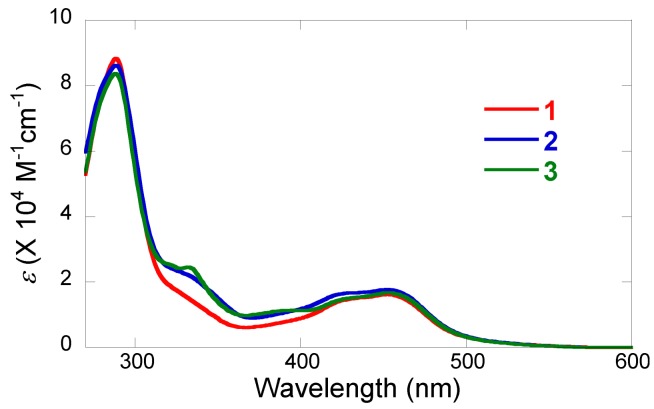
Absorption spectra of [**1**](PF_6_)_2_·3H_2_O (red line), [**2**]Cl·6H_2_O (blue line), and [**3**](PF_6_)_2_·2H_2_O (green line) in DMF at 20 °C in air.

The spectroscopic and photophysical data of **1**-**3** are listed in Table 1. UV-visible absorption spectra of **1**-**3** in DMF are shown in Figure 1. 

**Table 1:** Absorption and emission properties of **1**-**3**.^a^

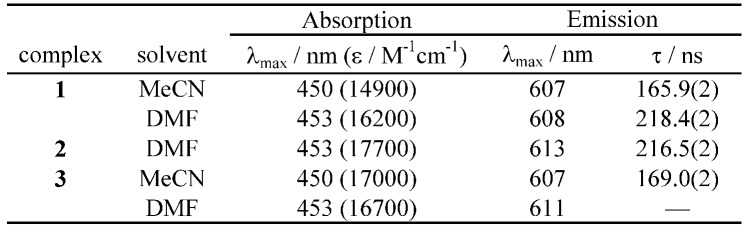

^a ^Measured in air at room temperature

Each complex displays a strong absorption band in the visible region at *ca*. 450 nm, unambiguously assigned to the metal-to-ligand charge transfer (MLCT) transition, d(Ru) → π*(bpy). No significant difference is seen in the spectra of **1**-**3** at the π → π* transitions around 300 nm. On the other hand, the absorptivities of **2** and **3** around 300-400 nm are higher than that of **1**, obviously due to the overlap of the MLCT band of the Pt(ppy)ClX moiety (X = Cl^-^ or DMSO) at these wavelengths [12,27,28].

**Figure 2:**
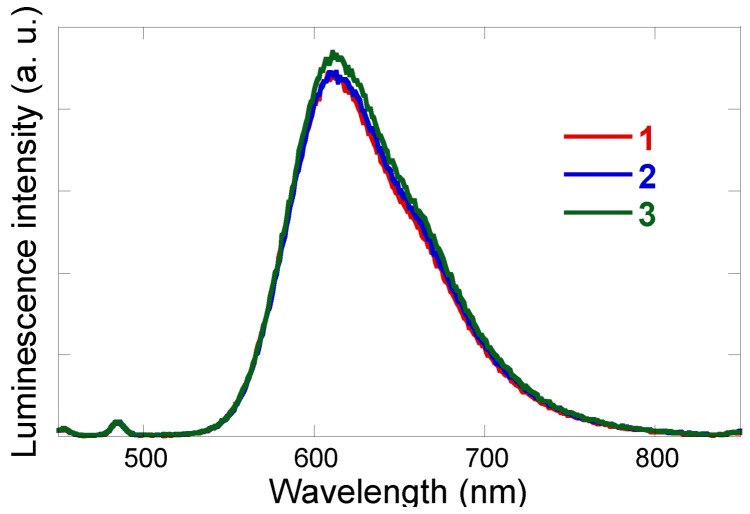
Luminescence spectra of [**1**] (PF_6_)_2_·3H_2_O (red line), [**2**]Cl·6H_2_O (blue line), and [**3**](PF_6_)_2_·2H_2_O (green line) in DMF at 20 °C in air, where the excitation wavelength was fixed at 425 nm and all the solutions had an equal absorbance at 425 nm (0.1).

Figure 2 shows the steady-state emission spectra of **1**-**3**, where the photon flux absorbed by each system is controlled as equal, by giving a common absorbance at the excitation wavelength. The luminescence intensity at *ca*. 610 nm for DMF solutions of **2** and **3** are similar to that of the precursor complex **1**. The emission decay profiles of **1**-**3** in air at room temperature all show a good fit to a monoexponential function. 

The emission lifetimes of **2** and **3 **are same as that of the precursor complex **1** (see Table 1), indicating that the triplet character of the [Ru(bpy)_2_(phen)]^2+^ unit in these Ru(II)Pt(II) dimers is preserved even after the platination. We recently pointed out that the platination-induced quenching ratio (the luminescence intensity ratio of the platinated product and the non-platinated precursor compound) is correlated with the photo-hydrogen-evolving activity of the molecular catalysts [10,22,23,24]. These results suggest that the intramolecular electron or energy transfer is not enhanced at all in these new Ru(II)Pt(II) dimers **2** and **3**, implying that desirable photoinduced intramolecular electron transfer leading to HER may not be enhanced in these systems.

### 2.3. Electrochemistry

**Figure 3:**
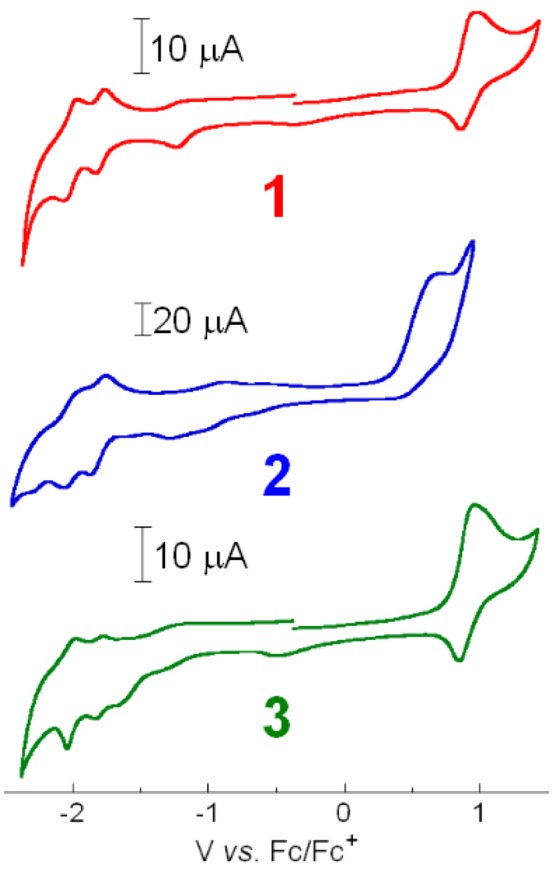
Cyclic voltammograms of [**1**](PF_6_)_2_·3H_2_O (red), [**2**]Cl·6H_2_O (blue) and [**3**](PF_6_)_2_·2H_2_O (green) in either 0.1 M TBAP/dry MeCN (**1**, **3**) or 0.1 M TBAP/dry DMF (**2**), recorded at a scan rate of 100 mVs^-1^.

The redox potentials for the electrochemical processes observed for **1**-**3** in either MeCN or DMF are summarized in Table 2. The cyclic voltammograms for **1**-**3** are shown in Figure 3. 

**Table 2:** Electrochemical data^a^ of **1**-**3** and **A**-**C**.

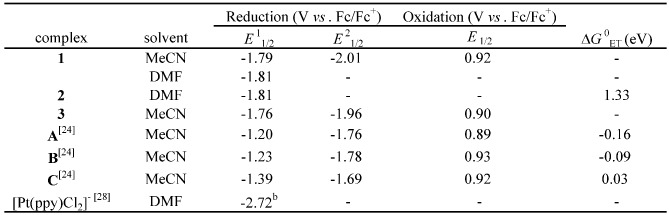

^a ^Determined at 20 °C with 0.1 M TBAP as the supporting electrolyte; scan rate 100 mVs^-1^; ^b ^This is converted from the value reported in V *vs*. SCE (*E*_1/2_ = -2.29 V *vs*. SCE) [28], where conversion was made using *E*(Fc/Fc^+^) = *E*(SCE) – 0.433 V [31].

The Ru(II)/Ru(III) couple in the non-platinated precursor complex **1** is observed at 0.92 V *vs*. Fc/Fc^+^. Upon platination of **1** into **3**, the Ru(II)/Ru(III) couple is slightly shifted to the negative side (*ΔE*_1/2_ = -0.02 V), as previously observed for **A**-**C** [24] (see Table 2). On the other hand, the first reduction peak for **1**-**3** occurs at around -1.8 V *vs*. Fc/Fc^+^ (Table 2), indicating that the first reduction of **1**-**3** occurs at the bpy or phen coordinated to the Ru(II) ion. Importantly, our recent studies on the related Ru(II)Pt(II)-based molecular devices **A**-**C** revealed that the first reduction must occur at the bpy bound to the Pt(II) ion and must be positioned at a potential more positive than *ca*. -1.2 V *vs*. Fc/Fc^+^ in order to serve as an active photo-hydrogen-evolving molecular device [24]. This was reasonably explained by comparing the driving forces for the intramolecular electron transfer from the [Ru*(bpy)_2_(phen)]^2+^ moiety to the π*(bpy attached to Pt) orbitals. As summarized in Table 2, molecular devices **A** and **B** exhibit activity towards photoinduced HER because of their downhill characters for the photoinduced electron transfer process, while **C** does not due to the uphill character (Δ*G*^0^_ET_ = 0.03 eV). On the other hand, since the reduction at the ppy bound to Pt does not occur at potentials close to -1.2 V *vs*. Fc/Fc^+^, the photoinduced electron transfer from the [Ru*(bpy)_2_(phen)]^2+^ moiety to the π*(ppy attached to Pt) orbitals is not thermodynamically favorable. It must be noted here that the present study was originally started in our laboratory prior to our approach to **A**-**C**, and thereby we were not aware of the fact that the intramolecular electron transfer from the [Ru*(bpy)_2_(phen)]^2+^ moiety to the π*(bpy or ppy attached to Pt) orbitals is a key factor to drive the photoinduced HER in these molecular devices. In other words, we decided to focus on the Pt(ppy)Cl_2_ moiety merely to improve the H_2_-evolving activity of the catalyst center when we started working on this study. Using the observed electrochemical and spectroscopic parameters, the driving force (Δ*G*^0^_ET_) for the intramolecular electron transfer from the ^3^MLCT excited state of the [Ru(bpy)_2_(phen)]^2+^ moiety to the tethering Pt(ppy) moiety is estimated based on the Rehm-Weller equation (1) [29]:

Δ*G*^0^_ET_ = *E*_ox_ – *E*_red_ – *E*_T_ – *e*^2^/*εd* (1)

where *E*_ox_ is the first oxidation potential of the Ru center, *E*_red_ is the first reduction potential of the ppy moiety bound to the Pt(II) ion, *E*_T_ is the energy of the ^3^MLCT exited state, *ε* is the dielectric constant of DMF, and *d* is the distance between the donor and acceptor. Since *ε* values are high for polar solvents like DMF, the last term is negligible. The value of *E*_T_ is estimated from a tangent to the high-energy side of the emission spectra of **2** in DMF (2.31 eV) [30]. In addition, from the reported reduction potentials of the ppy in [Pt(ppy)Cl_2_]^-^ (-2.72 V *vs.* Fc/Fc^+^; see also Table 2) [28] as well as the oxidation potential for the Ru(II)/Ru(III) couple for **1** (0.92 V *vs.* Fc/Fc^+^), the driving force for the intramolecular electron transfer from the [Ru*(bpy)_2_(phen)]^2+^ moiety to the π*(ppy attached to Pt) orbitals in **2** can be estimated as 1.33 eV, revealing that this is an amazingly uphill process. This was an obviously unexpected result for us, and the present study reconfirms the importance of having a negative value of Δ*G*^0^_ET_ to realize photo-hydrogen-evolving activity for such systems. 

### 2.4. DFT studies

**Figure 4:**
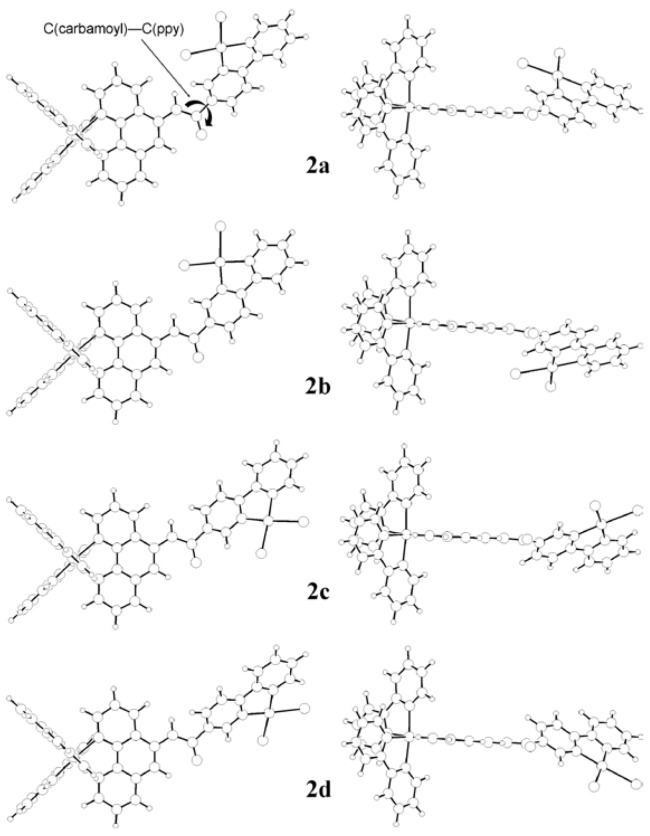
Optimized structures of **2** for the four possible conformers **2a**-**d** in water, computed at the B3LYP level of DFT using the LanL2DZ base set, using the PCM method.

To better understand the electronic structures of the new Ru(II)Pt(II) dimers, the structures of the compounds were computed by the DFT method. First, the four possible conformers of **2** were developed and the structures in aqueous media (polarizable continuum model; PCM) were fully optimized at the B3LYP level of DFT (conformers **2a**-**d** in Figure 4). The relative energies of structures **2a**-**d** are listed in Table 3, showing that the maximum shift is only 2.5 kcal/mol. 

**Table 3:** Relative energies and dihedral angles for the four possible conformers of **2**.

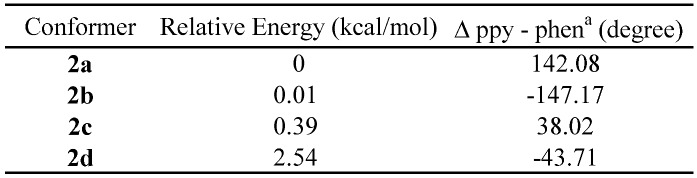

^a^Dihedral angles between the ppy and the phen planes

**Figure 5:**
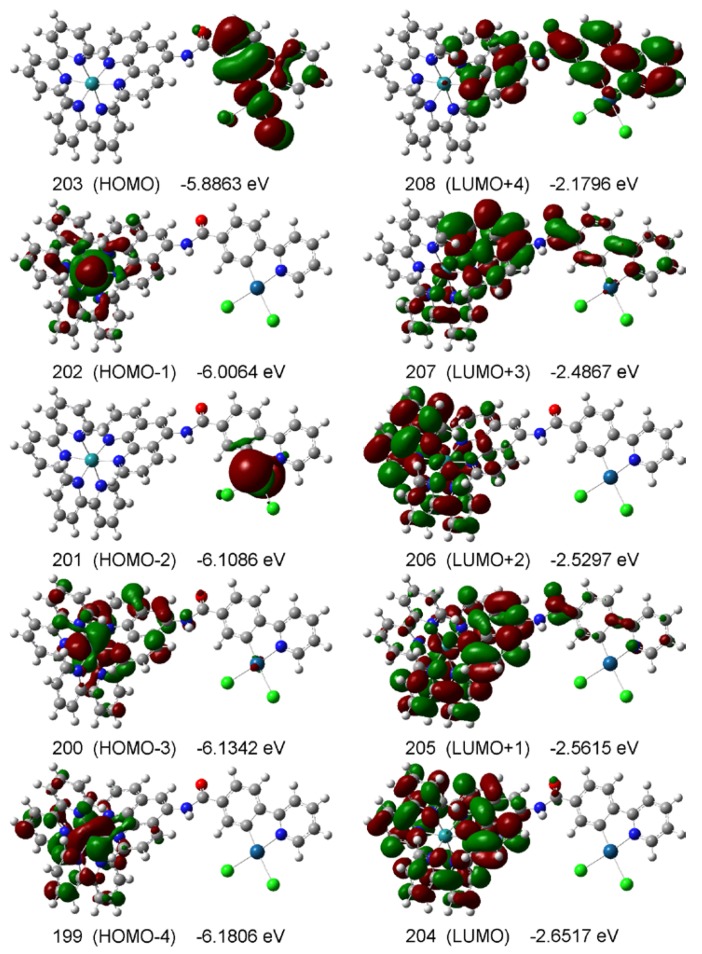
Frontier molecular orbitals of a fully optimized structure of conformer **2a** for **2** in water, where the structure was computed at the B3LYP level of DFT using the LanL2DZ base set, using the PCM method.

For the conformer having the lowest energy (*i.e.*, conformer **2a**), some of the relevant frontier molecular orbitals are depicted in Figure 5. The HOMO consists of p_z_(Cl), d_xz_(Pt), and π(ppy) orbitals, where the z axis is taken perpendicular to the Pt coordination plane and the x axis is take along the C(ppy)−Pt−Cl axis. These orbitals are coupled in such a manner that both the C(ppy)−Pt and Pt−Cl bonds bear the anti-bonding characters. The HOMO-2 corresponds to the d_z_2(Pt) orbital, while the HOMO-1, HOMO-3, and HOMO-4 the d(Ru) orbitals. On the other hand, the major contributions to the four lowest unoccupied MOs (*i.e.*, LUMO-LUMO+3) are either π*(bpy) or π*(phen) orbitals. The LUMO+4, which is *ca*. 0.47 eV higher in energy than the LUMO, is the first lowest unoccupied MO with a major contribution from the π*(ppy) orbitals. These confirm that the first reduction of **2** and **3** occurs at either bpy or phen rather than at ppy. 

### 2.5. Photolysis experiments

**Figure 6:**
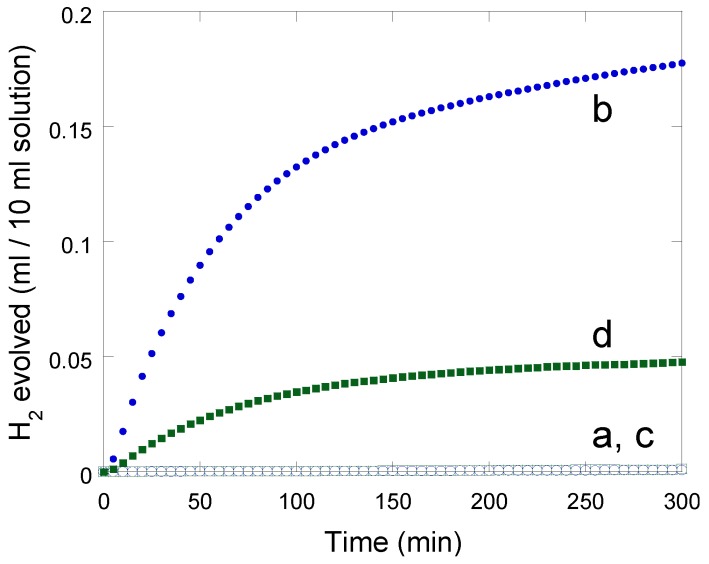
Photochemical H_2_ production from an aqueous acetate buffer solution (0.03 M CH_3_CO_2_H, 0.07 M CH_3_CO_2_Na, pH 5.0, 10 mL) containing 30 mM EDTA in the presence of (a) 0.1 mM [**2**]Cl·6H_2_O, (b) 0.1 mM [**2**]Cl·6H_2_Oand 2 mM MV(NO_3_)_2_, (c) 0.1 mM [**3**](PF_6_)_2_·2H_2_O, and (d) 0.1 mM [**3**](PF_6_)_2_·2H_2_Oand 2 mM MV(NO_3_)_2_.

In order to evaluate the photo-hydrogen-evolving activity of the new Ru(II)Pt(II)-based compounds **2** and **3**, the amounts of H_2_ evolved during the photoirradiation of the compounds in aqueous media were monitored by gas chromatography under the experimental conditions same to those adopted in our previous studies on **A**-**C** [13,24]. The pH of each solution was adjusted at 5.0 (acetate buffer) and EDTA was added as a sacrificial electron donor, as previously described. As discussed above, **2** and **3** do not drive the photoinduced HER at all (see Figure 6, lines a,c). However, important observations are that an effective amount of H_2_ evolves in the presence of methylviologen (MV^2+^) (see Figure 6, lines b,d). The total amount of H_2_ evolved after 300-min irradiation was 0.18 mL for the EDTA/**2**/MV^2+^ system and 0.05 mL for the EDTA/**3**/MV^2+^ system. The turnover number (TON), estimated from the total amount of H_2_ evolved and the amount of Ru(II)Pt(II) dimer employed (1 μmol) was TON = *ca*. 8 (after 5 h) for the EDTA/**2**/MV^2+^ system and TON = *ca*. 2 (after 5 h) for the EDTA/**3**/MV^2+^ system. The former is effectively higher than the value of TON = 2.4 (after 10 h) reported for the EDTA/**A** system [13]. These results clearly indicate that both **2** and **3** involve an active photosensitizer unit, [Ru(bpy)_2_(phen)]^2+^, as well as a catalyst center active enough to promote the HER. It is thereby reasonable to consider that the ^3^MLCT excited state of the [Ru(bpy)_2_(phen)]^2+^ unit in **2** or **3** is *intermolecularly* quenched by the coexisting electron relay (MV^2+^) to once afford MV^+^, which is then consumed to drive the HER as a dark reaction, as recently reported by the authors [32]. Moreover, photoirradiation of a system consisting of EDTA, [Ru(bpy)_3_]^2+^, and a Pt(II)-based catalyst does not lead to HER [7,8], which suggested that [Ru(bpy)_3_]^+^ {*i.e.*, [Ru(bpy)_2_(bpy^-^**·**)]^+^} is not given during the photolysis (reductive quenching of [Ru*(bpy)_3_]^2+^ by EDTA does not proceed under those conditions). This further explains the reason why no hydrogen evolves with use of the EDTA/**2** and EDTA/**3** systems, in which oxidative quenching of the [Ru*(bpy)_3_]^2+ ^ moiety by the H_2_-evolving catalyst site cannot be promoted in an intramolecular fashion.

## 3. Conclusions

The new heterodinuclear Ru(II)Pt(II) complexes **2** and **3** possessing Pt(ppy)ClX units have been synthesized and characterized. Unfortunately, these dimers do not exhibit catalytic activity as photo-hydrogen-evolving molecular devices in a single-component fashion due to the largely uphill character for the photoinduced electron transfer process. This study reconfirms the importance of controlling the driving force for the electron transfer from the photosensitizing center to the catalyst center to promote the HER within such a photosynthetic molecular device. We now realize that the molecular design strategy must, at the same time, fulfill two factors; one is to improve the H_2_-evolving activity of the Pt(II)-based moiety, while the other is to have at least one electron acceptor unit in the close vicinity of the catalyst center. Such attempts are now in progress in our laboratory.

## 4. Experimental

### 4.1. Materials and measurements

K_2_PtCl_4_ was purchased from Tanaka Kikinzoku Kogyo and used as received. All solvents and reagents were of the highest qualities available and were used as received without further purification. *N*-(1,10-phenanthrolin-5-yl)-4-(2-pyridyl)benzamide (H**L**) [25], (Bu_4_N)_2_[PtCl_4_] [27], *cis*-RuCl_2_(bpy)_2_ [33], and *cis*-PtCl_2_(DMSO)_2_ [34] were synthesized as previously described.

UV-visible spectra were recorded on a Shimadzu UV-2450 spectrophotometer. IR spectra were recorded on a Perkin Elmer Spectrum One equipped with a diamond ATR (attenuated total reflection) system. ^1^H-NMR spectra were measured on a JEOL JNM-AL300 spectrometer. Luminescence spectra were recorded on a Shimadzu RF5300PC spectrofluorophotometer. Emission lifetime data were determined using a customized apparatus equipped with an Iwatsu DS-4262 digital oscilloscope and a Hamamatsu R928/C3830 photomultiplier tube coupled to a Horiba H-20-VIS grating monochromator. The excitation source was an N_2_ laser (337 nm) (Usho KEN-1520). Electrochemical measurements were carried out in argon-purged acetonitrile (MeCN) or *N*,*N*-dimethylformamide (DMF) solutions at a sweep rate of 100 mVs^-1^ using a Pt disk working electrode, a Pt wire counter electrode, and an Ag wire reference electrode (490 mV *vs*. NHE). The supporting electrolyte was 0.1 M tetra(*n*-butyl)ammonium perchlorate (TBAP). ESI-TOF mass spectra (ESI-TOF MS) were recorded on a JEOL JMS-T100LC mass spectrometer. All the ESI-TOF MS measurements were performed in the positive-ion mode, at a cone voltage of 20 V, and the mobile phase was acetonitrile or methanol. Typically, a solution of each sample was introduced onto the spectrometer at a flow rate of 10 μL min^-1^ using a syringe pump.

### 4.2. DFT calculations

Density functional theory (DFT) calculations were performed using the Gaussian 03 package of programs [35]. The structure was fully optimized using the B3LYP method which uses hybrid Becke's three-parameter exchange functional [36] with the correlation energy functional of Lee, Yang and Parr [37]. Calculations were performed using the standard double-ζ type LanL2DZ basis set [38,39,40] implemented in Gaussian 03, without adding any extra polarization or diffuse function. All the calculations were performed using the polarizable continuum model (PCM) [41] to compute the structures in aqueous media. We experienced that the results obtained by the PCM method are clearly different from those obtained for the structures in their gaseous states, and is considered as more realistic with respect to the energy level of the molecular orbitals. 

### 4.3. Photolysis experiments

Photochemical hydrogen production from water was analyzed using the automatic H_2_ monitoring system developed in our group. In this system, a continuous flow of Ar (10.0 mL min^-1^, controlled by an STEC SEC-E40/PAC-D2 digital mass flow controller) was bubbled through each photolysis solution (10 mL) contained in a Pyrex vial (*ca*. 20 mL). The vent gas from the vial was introduced into a 6-way valve which allowed the automatic injection of the sample gas onto a gas chromatograph (Shimadzu GC-14A equipped with a 5Å molecular sieve column of 2.5 m × 3 mm *i.d.*, thermostatted at 30 °C). The injection of sample gas was driven by a control software operating on a Windows system. The signal output from the thermal conductivity detector was also monitored using the same software. Photolysis solutions were deaerated with Ar for at least 30 min prior to the photolysis. The photoirradiation was carried out by an Ushio Xe short arc lamp UXL500D-O (operated at 350 W). The photolysis vial was immersed in a water bath thermostatted at 20 °C to remove IR radiation and to eliminate the temperature effect.

### 4.4. Syntheses

[**1**]*(PF_6_)_2_·3H_2_O*. A solution of *cis*-RuCl_2_(bpy)_2_·3H_2_O (0.13 g, 0.25 mmol) and H**L**·1.5H_2_O (0.094 g, 0.23 mmol) in ethanol (10 mL) was refluxed for 15 h. After the solution was cooled down to room temperature, the solution was filtered for the removal of insoluble materials. Then, water (15 mL) was added to the filtrate, followed by removal of ethanol by evaporation. To this solution was added a saturated aqueous solution of NH_4_PF_6_ (*ca*. 1 mL). The orange powder deposited was collected by filtration and dried *in vacuo*. Yield: 0.24 g (85%). Calculated for C_44_H_38_N_8_O_4_P_2_F_12_Ru: C, 46.41; H, 3.38; N, 9.73. Found: C, 46.34; H, 3.07; N, 9.73; ^1^H-NMR (300.53 MHz, DMSO-d_6_), ppm: 11.04 (s, 1H), 8.90-8.80 (m, 6H), 8.74 (d, 1H, *J* = 4.8 Hz), 8.62 (s, 1H), 8.34 (d, 2H, *J* = 8.3 Hz), 8.25-8.07 (m, 8H), 8.00-7.85 (m, 5H), 7.61-7.57 (m, 5H), 7.47-7.36 (m, 3H); ESI-TOF MS: m/z 395.00 [M - 2PF_6_ - 3H_2_O]^2+^; FT-IR (cm^-1^): 3649 (w), 1675 (m), 1630 (m), 1605 (m), 1587 (m), 1528 (m), 1467 (m), 1434 (m), 1425 (m), 1385 (m), 1314 (m), 1276 (m), 1254 (m), 1162 (m), 1015 (m), 836 (s), 802 (m), 760 (s), 731 (m), 557 (s).

[**2**]*Cl·6H_2_O*. [**1**](PF_6_)_2_·3H_2_O (0.057 g, 0.050 mmol) was dissolved in a 1:1 H_2_O-ethanol mixture and was treated with an anion-exchange resin (Amberlite IRA-400) followed by removal of solvents to give the dichloride salt of the complex ([**1**]Cl_2_) in a quantitative yield. The residue (*i.e.*, [**1**]Cl_2_) and (Bu_4_N)_2_[PtCl_4_] (0.050 g, 0.061 mmol) in methanol (12 mL) was then sealed in a pressure-resistant vial and was stirred at 140 °C for 4 h. After the solution was cooled down to room temperature, the solution was filtered for the removal of insoluble materials (*Caution!* Do not open the vial while it is hot, since the solution splashes out because of the violent boiling phenomenon upon a sudden decrease in pressure.). The filtrate was concentrated by evaporation under reduced pressure to a total volume of *ca*. 1 mL. Addition of acetone (20 mL) to the solution resulted in prompt deposition of the product as an orange powder, which was collected by filtration and dried *in vacuo*. Yield: 0.022 g (37%). Calculated for C_44_H_43_N_8_O_7_Cl_3_RuPt: C, 44.10; H, 3.62; N, 9.35. Found: C, 44.18; H, 3.60; N, 9.38; ESI-TOF MS: m/z 1054.85 [M - Cl - 6H_2_O]^+^; FT-IR (cm^-1^): 3369 (m), 3073 (m), 1650 (m), 1627 (m), 1602 (m), 1515 (m), 1480 (m), 1463 (m), 1445 (m), 1423 (m), 1382 (m), 1312 (m), 1270 (m), 1245 (m), 1160 (m), 1108 (m), 1065 (m), 882 (m), 762 (s), 725 (m), 417 (m).

[**3**]*(PF_6_)_2_·2H_2_O*. A solution of [**1**](PF_6_)_2_·3H_2_O (0.057 g, 0.050 mmol) and *cis*-PtCl_2_(DMSO)_2_ (0.025 g, 0.060 mmol) in methanol (10 mL) was sealed in a pressure-resistant vial and was stirred at 140 °C for 4 h. After the solution was cooled down to room temperature, the solution was filtered for the removal of insoluble materials (*Caution!* Do not open the vial while it is hot, since the solution splashes out because of the violent boiling phenomenon upon a sudden decrease in pressure.). By repeating gradual removal of ethanol by evaporation and by gradual addition of water, the major solvent was exchanged from ethanol to water until the total volume of the solution became *ca*. 10 mL. Addition of a saturated aqueous solution of NH_4_PF_6_ (*ca*. 0.5 mL) to the former solution resulted in prompt deposition of the product as an orange powder, which was collected by filtration and dried *in vacuo*. Yield: 0.050 g (71%). Calculated for C_44_H_41_N_8_O_4_ClSP_2_F_12_RuPt: C, 38.81; H, 2.90; N, 7.87. Found: C, 38.64; H, 2.75; N, 7.69; ^1^H-NMR (300.53 MHz, DMSO-d_6_), ppm: 10.04 (s, 1H), 9.69 (d, 1H, *J* = 5.7 Hz), 9.22-9.18 (m, 2H), 8.96 (d, 1H, *J* = 4.1 Hz), 8.89-8.80 (m, 5H), 8.50 (dd, 1H, *J* = 5.1 Hz, *J'* = 1.1 Hz), 8.38 (dd, 1H, *J* = 5.18 Hz, *J'* = 1.2 Hz), 8.30-8.14 (m, 8H), 8.00-7.88 (m, 6H), 7.68-7.61 (m, 3H), 7.45-7.40 (m, 2H), 3.71(d, 6H, *J* = 3.1 Hz); ESI-TOF MS: m/z 548.97 [M - 2PF_6_ - 2H_2_O]^2+^; FT-IR (cm^-1^): 3649 (w), 1680 (m), 1629 (m), 1606 (m), 1524 (m), 1483 (m), 1466 (m), 1447 (m), 1424 (m), 1380 (m), 1315 (m), 1271 (m), 1246 (m), 1163 (m), 1135 (m), 1123 (m), 1025 (m), 835 (s), 760 (s), 730 (m), 556 (s), 444 (m), 421 (m).
